# Preferences of overweight and obese patients for weight loss programmes: a discrete-choice experiment

**DOI:** 10.5334/ijic.1113

**Published:** 2013-09-20

**Authors:** Axel Mühlbacher, Susanne Bethge

**Affiliations:** Health Economics and Healthcare Management, Institute Health Economics and Healthcare Management, Neubrandenburg, Germany; Institute Health Economics and Healthcare Management, Neubrandenburg, Germany

**Keywords:** overweight and obesity, patient preferences, discrete-choice experiment, stated preferences, integrated care, coordinated care

## Abstract

**Introduction:**

Obesity is associated with increased risk of morbidity and mortality and also appears to have an adverse effect on health-related quality of life. Though advances in obesity therapy and rehabilitation can be observed, the long-lasting outcome is dissatisfying to most of the patients and, therefore, the whole health care system.

**Theory and methodology:**

The study aims to identify key attributes of coordinated weight loss programmes and elicit patients’ preferences for overweight and obesity therapy in rehabilitation programmes. A self-administered survey measuring attitudes and preferences was conducted in Germany in 2009. Discrete-choice experiment scenarios were developed using a fractional factorial design and results analysed using a random effects logit model.

**Results:**

*N*=110 patients completed the questionnaire, 51.82% of these were male, the mean age was 53.05 years and mean body mass index was 33.54 kg/m2 (SD 7.73). A total of 823 choices could be included in the final estimation. The most important aspects for the respondents’ selection were care coordination (coefficient 1.473; SE 0.185) and individual therapy (coefficient 1.446; SE 0.188). The aspect ‘infrastructure of care’ (coefficient 0.570; SE 0.175) was less relevant. All attributes led to significant coefficients.

**Conclusion:**

Patients value coordination of care and individual therapy most highly. So weight reduction therapy should enable patients to receive a structured, coordinated and interpersonal therapy that is tailored to their personal needs, behaviour and circumstances. Patients are willing to forego infrastructure quality in favour of better coordination and structure in their therapy.

## Background: treatment goal is sustained lifestyle change

Overweight and obesity have reached epidemic proportions in Germany [1]. Due to the high prevalence of comorbidities, the prevention and therapy of overweight and obesity represent major challenges to our health care system [2,3]. Overweight and obesity reduce life expectancy and have a negative effect on quality of life, and over a million of deaths can be attributed to the conditions. Estimates indicate that overweight and obesity generate up to 60% of health costs for adults in the European Union; in addition, they lead to at least twice that amount of indirect costs (through loss of life and productivity or the associated income) [4]. As in the USA, in Germany, overweight has also become one of the most significant health problems and cost factors, and despite major investment in research, treatment and prevention, there is no sign of a reversal in this trend [5–12].

In view of the disappointing results of the therapy approaches used to date, future obesity treatments must break fresh ground and new ways of treatment and treatment coordination are needed [13]. Coordinated programmes refer to the meaning of the managed care approaches. It should reflect that it is not an effort a patient is making on his own rather a coordinated (managed) weight reduction therapy. One major success factor within this coordinated treatment is the implementation of ‘a sustained lifestyle change’ [14]. The initiation of a lifestyle change requires the motivation of the patient. The therapist can only motivate the patient if he knows the factors that are patient-relevant. Otherwise, lack of compliance, repeated discontinuation of therapy, other failures and the associated general hopelessness can occur [15,16]. Depending on the measure and the method of data collection, lack of compliance in obesity treatment is reported in 50–83% of patients [17].

If it is assumed that patients try to maximise their benefit, then the consideration of individual motivation is an essential criterion for the success of treatment. All potential users of health care services only maximise their benefit if the health care services offered are geared towards patients’ individual needs and take account of their preferences. The preferences are the result of individual expectations of benefit. Anyone who knows the preferences can engender lasting changes of behaviour. The therapist should know the preferences of patients/patient groups to be able to offer a correspondingly effective range of services. Despite the obvious urgency of effective treatment programmes that incorporate patient's needs, there is no evidence that treatment programmes are oriented specifically towards individual preferences.

The focus of this study was the identification and quantification of the preferences of overweight and obese rehabilitation patients. The question arising here was which therapy attributes determine patient benefit, motivation and the quality of a treatment from the patient's perspective and to what extent?

For the patient's perspective, comprehensive objective benefit parameters (medical outcomes), perceived patient's benefit and satisfaction factors, the risk (respective to damage) and the costs (individual and social resource consumption) must be taken into account (see also the model on utilisation of health care services [18]). Often, the success of rehabilitation is affected considerably by the success of weight reduction, for instance, in patients with coronary heart conditions or orthopaedic complaints. The research results are intended to be used in the preventative, curative and, especially, in the rehabilitation sectors of obesity treatment. The ascertained preferences should also be applied to system-related topics as for the design, realisation and evaluation of coordinated and integrated care programmes, as patient-centeredness is seen as a major success factor. (It should be mentioned that the study focuses on patient's preferences within rehabilitation facilities and setting specific characteristics. Therefore, outcome parameters as well as personal characteristics as evaluated in other studies were left out of this experiment [19,20].)

## Methodology

### Theoretical background

Patient preferences are increasingly being elicited in health economics using discrete-choice analysis (or discrete-choice experiment) [21]. The discrete-choice experiment is the choice-based form of conjoint analysis which was made possible by the theoretical work of Lancaster [22] and McFadden [23]. The implementation of discrete-choice methodology offers numerous practical advantages so that it has already developed into a popular form of preference measurement in health economics [21]. This is also evident from the steadily increasing number of discrete-choice Experiments published in the public health service for various indications, such as Attention-Deficit Hyperactivity Disorder (ADHD) [24], multiple sclerosis [25] and type II diabetes [26], as well as to evaluate integrated care programmes [27–30].

This method of evaluating preferences is based on the demand theory developed by Lancaster (1966) [22], according to which the benefit that a product or a service (weight reduction programme) generates for consumers (or demanders or patients) depends upon the characteristics of the product and the attitudes of the consumer. The benefit of a therapy for weight loss depends upon the characteristics of the therapy: effectiveness, reduction of symptoms or side effects, etc. [31].

The discrete-choice method assumes a stochastic utility function (random utility function) (see McFadden 1974 and 2001 [32,33]). The core statement of the *random utility model* is that not all the determinants affecting benefit or utility are perceptible to the observer so that the choices of the respondents are not predictable. The determinants which are not perceptible to the observer appear to the observer as random distortions in the utility function [34]. Preferences are thus to be seen as a random factor. Yet it is also the case that individuals make considered and systematic choices and that these are not based on random decisions [31]. Further details to the method of discrete-choice experiment, assumption of the utility function and distribution of utility components can, for example, be found in Ref. [21,35–37].

### The study design

#### Establishment of relevant product attributes

In order to be able to anticipate and explain the decisions of patients or insured parties for a medical service, e.g. a weight loss therapy, it is necessary to elicit all the attributes relevant to the decision of the particular target group (obese patients) first. For a measure aimed at sustained weight reduction, these could be, e.g. level of activity, nutritional demands, duration of the measure, design of the treatment, and side effects in the case of drug support or even price (or supplemental payment required). In principle, there are three methods to ascertain the relevant attributes, which are not mutually exclusive and can also usefully be applied in parallel:

- Assessment of not only scientific literature and case studies but also reports of persons affected (e.g. patient reports).
- Group discussions with the target group, particularly in the context of focus groups.
- Personal interviews with not only members of the target group but also experts (e.g. doctors or pharmacists who have contact with the target group in a relevant context) or even standardised interviews, e.g. in the form of repertory grid methods with overweight or obese patients.

The attributes identified as being relevant to the survey target group can be either procedural or result-related attributes of a medicinal product or a service. It is precisely the simultaneous evaluation of procedural and output characteristics that represents one of the major advantages of the discrete-choice method. In addition, the attributes need not necessarily be describable in quantitative terms (e.g. duration of effect in hours), qualitative classifications are also possible (e.g. friendliness of clinic staff). Within a preliminary study, literature research and focus groups were conducted to identify patient-relevant attributes within weight reduction therapies in rehabilitation facilities. The detailed results and descriptions regarding the qualitative study-phase are presented within a preliminary article [38].

The patient-relevant attributes identified in this first part of the study have been structured, classified and summarised using psychometric testing (e.g. factor analysis) and corresponding validation (e.g. reliability testing). The developed eight dimensions (factors), representing the patient-relevant decision criteria form the basis for the attributes used in this discrete-choice experiment.

#### Selection of choice sets and conduct of the experiment

A choice set consists of two or three actual hypothetical treatment alternatives between which the subjects can or must choose. The choice set displays a combination of the eight identified attribute descriptions. Depending on the application, it may also make sense to offer the respondents a no-choice alternative. We have decided against the option of a no-choice alternative in this study and have instead used a ‘forced choice set’ were participants had to choose between two possible therapy alternatives [39].

**Figure 1.**
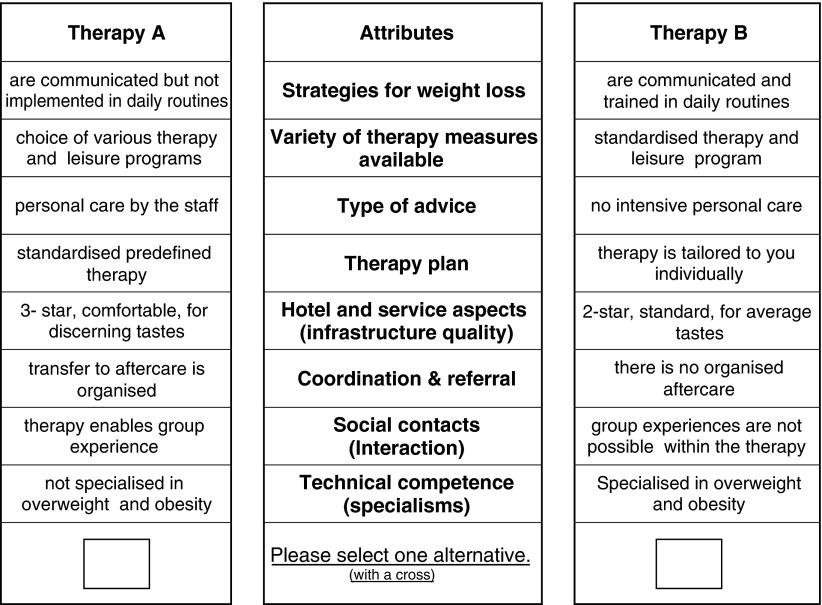
Example of a choice set (out of the 12 presented choice tasks).

The attributes and attribute manifestations of each product combination were displayed alongside to each other so that the subjects in the experiment could see the differences of the alternatives available at a glance. This enabled the participants to better weigh up the various aspects according to their own preferences. Figure 1 exemplarily shows how a choice set of this study looked.

Each respondent was presented with the choice sets of the possible weight loss measures one after another and required to make a discrete choice in each case (in the example in Figure 1 for the therapy option A or therapy option B). Hence, the respondents compared two alternatives and selected the one which generated the greatest benefit for them or the highest expectation of benefit (utility maximisation).

As it was not possible to include all available attribute combinations out of the identified attributes, an experimental design with a reduced number of choice tasks needed to be constructed. The reduced (or factorial) design should contain the subsection of alternatives which best represents the complete set of alternatives (full design). The goal was to minimise the loss of information inherent in reducing the design. When making the selection, it must be ensured that no information is lost [40,41].

A specific form of the reduced design is represented by what is called the orthogonal design in which all rateable effects are uncorrelated. Orthogonal designs are also categorised according to their dispersal (for more details on this see [42]). This study used such a reduced design on the basis of an orthogonal main effects plan. As a basic design for the choice task, an orthogonal main effects plan was generated using SPSS®17, and the associated sets were then created using the fold-over method.

#### Evaluation of the survey

If *N* persons participate in a discrete-choice experiment and each person makes *E* selections, there are N*E selections which can be statistically evaluated to analyse the influence of the various attributes on the selection. Prior to the analysis, however, the data must be recoded. Depending on the number of attribute manifestations, an effect coding must be performed or a dummy coding may be sufficient [42]. In the later evaluation, a dummy coding was used due to the binary manifestations of the level. The estimation of the utility function was done using the maximum likelihood method [43]. The estimated utility function is expressed as a constant and as a coefficient for each product attribute. The level of the coefficients of the various attributes does not enable direct statements on the influence of the described variables (attributes) on the selection decisions, but on the relationship of the various coefficients to one another.

## The results: patient preferences in weight reduction programmes

Based on the results of the preliminary qualitative study [38], a discrete-choice questionnaire was created. This was intended to be completed by rehabilitation patients themselves. It was, therefore, important to reduce the complexity of the questionnaire as far as possible. In December 2009, a preliminary test was carried out (*N*=7) to check the methodology and practicality of the questionnaire.

Overall, the evaluation showed that from the patients’ point of view, the questions were clear and understandable. In addition, the sequence of the questionnaire sections was changed. Because of the attention curve, the actual experiments were put at the beginning of the questionnaire and the socio-demographic and health status questions relegated to the end. The questionnaire used in the main survey was divided into four sections.

Section A

- Introduction to the attributes with ranking of the most important items, direct importance and status quo survey of the attributes and manifestations used later in the discrete-choice experiment.

Section B

- Discrete-choice experiment to measure patient preferences with 12 pairs and a control pair with 8 characteristics each.
- Request for weighting of the decision and trade-off of the decision against status quo.
- Ranking of therapy attributes.

Section C

- Socio-demographic parameters: age and gender, school-leaving qualification, occupation, income.
- Questions associated with overweight and obesity: weight, height, waist and hip circumference, causes of increased weight, eating and exercise habits.

Section D

- Health status (SF12v2, Version SOEP).

**Figure 2.**
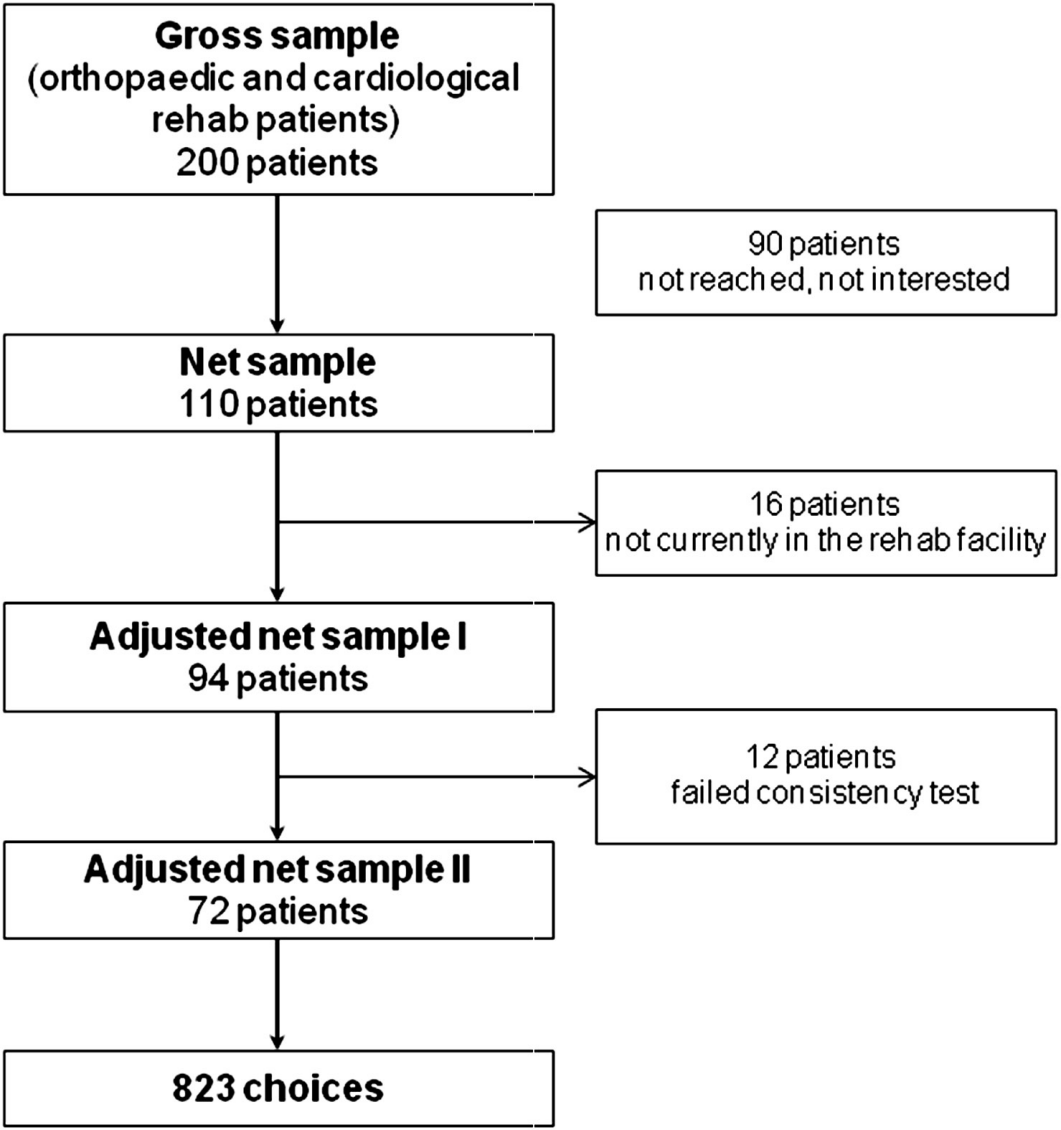
Process of respondents’ inclusion in the evaluation.

The analysis was based on the adjusted net sample of 72 participants (*N*=110 original net sample). All patients had to be overweight, older than 18 years, within the rehabilitation facility for at least 6 days and had given written informed consent to participate. As the experiment was limited to filling out a questionnaire an ethical approval was not necessary. Respondents who were not in a rehabilitation facility when the survey was completed were excluded from the preference calculation (*N*=16). Furthermore, participants were excluded (*N=*12) who failed the consistency test since it had to be assumed that these respondents were not capable of performing realistic trade-offs (Figure 2).

The main survey was carried out from March to April 2009, using pencil-and-paper questionnaires in three rehabilitation facilities. The socio-demographic evaluation produced values which are described in Table 1.

The individual dimensions of the discrete-choice experiments were explained to the respondents in the first section (A) of the questionnaire using what is known as ‘cheap talk’. This served to attune the patients directly to the relevant dimensions and clarify the areas included [44]. The explanations on the dimensions in the questionnaire for the patients were as follows:

1. Coordination and referral

Characteristics include, e.g.:

- availability of outpatient aftercare (the rehabilitation facility offers you the option of outpatient aftercare there)
- organisation of aftercare
- collaboration of the facility with general practitioners (GPs)

2. Individualised therapy planning

The characteristics of individualised therapy planning include, e.g.:

- explanations of the causes and implications of the condition
- analysis of your exercise routines
- individual exercise options

3. Interpersonal care

This includes, e.g.:

- staff motivation
- friendliness of staff members
- respectful attitude to you as the patient

4. Technical competence

The orientation of the facility includes, e.g.:

- the specialisms of the treating doctors
- the competence and experience of the doctors
- individual therapy

5. Social interaction

Characteristics include, e.g.:

- exercise in a group
- group therapy
- discussion groups

6. Knowledge

Characteristics include, e.g.:

- shopping training
- preparation of a detailed nutrition plan
- predefined portions for meals in the rehabilitation facility

7. Varied range of therapy options available (access)

These options could be, e.g.:

- wide variety of therapy options
- choice of various leisure activities (exercise options, cultural evening activities)
- support from your medical insurer

8. Hotel and service aspects (infrastructure quality)

These include, e.g.:

- room size
- room amenities
- size of the facility

In total, 72 respondents were included in the final evaluation. The coefficients were calculated using Stata^©^9 by means of logit estimation and with dummy coding. Statistically significant values were obtained for all attributes. The results are presented in Table 2.

## Discussion: patient orientation in weight reduction therapy

### Coordination and referral

Overweight and obese patients prefer continuous guidance, treatment and also aftercare. This attribute achieved the highest preference value (coefficient: 1.4736). In many cases, these patients have already gone through a ‘diet career’. Numerous attempts to lose weight on their own have generally failed. In order to achieve sustained weight loss, they need small consecutive treatment goals which should be adapted and supplemented depending on how they are received and the success rate. A long-term treatment strategy thus also includes an appropriate timescale.

At the same time, continuous aftercare is highly significant since the main goal of treatment is to sustain the weight loss achieved during rehabilitation in the long term. The so-called ‘yo-yo effect’” is to be avoided. Patients prefer a coordinated follow-up treatment beyond their stay in the rehabilitation facility. This is an essential precondition to ensure sustained weight reduction.

Continuous care can ensure that patients receive optimal support throughout the protracted process of weight loss. Referral into monitoring programmes following on from actual therapy can also contribute significantly to sustained weight stabilisation. Long-term, continuous and comprehensive care is thus the subject of generally recognised treatment guidelines, e.g. those of the German Obesity Society [45]. In this connection, the study of Mata et al. [46] inferred that longer-term care or a longer period within weight loss programmes significantly increases the likelihood of permanent (sustained) weight loss. A clear preference for support, backing and help during therapy was also shown, inter alia, in the study conducted by Kayman et al. [47]. According to this, ‘relapse patients’ in particular want more backing and support during the weight loss process.

### Individualised therapy planning

Furthermore, it is of great significance for patients (second most important attribute, coefficient: 1.4468) that their personal situation and individual circumstances are considered. Consideration of individual exercise and nutritional behaviour, daily habits and individual daily routines and social framework conditions are elementary to successful therapy. Tailoring therapy to the individual living and peripheral circumstances of the patient is a decisive criterion. Respondents who were able to integrate the required measures into their everyday lives achieved the best and most sustained results [47]. It can be concluded that a standard therapy (‘one fits all’) is not what patients want, not what patients need and, therefore, cannot make a positive contribution to long-term motivation. Due to patients’ very different living circumstances and risk factors, therapy measures should be designed to be flexible so that they can take account of the living circumstances of the relevant patients.

### Interpersonal care

The personal care of the patient, individual addressing and interpersonal communication with the patients in the form of friendliness and approachability of the staff was identified as the third most important attribute (coefficient: 1.3473) within the relevant characteristics of weight loss therapy. In a process control study of weight loss interventions, it was shown that direct addressing with face-to-face interaction had a highly significant correlation with sustained weight loss and a major influence on achievement of therapy goals (compared with, i.e. Internet support or control groups without any sort of personal addressing) [48]. As a result of this study, it was also observed that the rate of participation over an 18-month trial period was highest in the face-to-face groups (compared to purely online communication or without intervention). This means that personal care leads to patients remaining within the intervention programme for longer, which in turn significantly improves the achievement and maintenance of weight loss. This emphasises the tremendous importance of individualised and interpersonal care.

### Technical competence

The guidance and support of trained specialist staff, therapists and appropriately specialised doctors was also identified as an important characteristic (coefficient: 1.2902). Potter et al. (2001) showed in their study that all the groups of patients surveyed demanded greater involvement from their doctors, particularly in the establishment of realistic goals and increased involvement in weight management programmes [49]. In this connection, the ‘National task force on the prevention and treatment of obesity’ also points out the importance of structural quality and unimpeded access. This also includes access to the health care system, explicitly to appropriately specialised health care facilities and specialists [50].

### Social interaction

Social interaction can be seen as the exchange of those affected with ‘like-minded persons’ (coefficient: 0.9608). This provides patients with the opportunity to compare notes, to share successes and create a group dynamic. The common ‘experience’ within the rehabilitation facility can be a powerful motivator and is consequently preferred by the respondents. In addition, social interaction can be seen as exchange and feedback from the patient's own social circle. It is important for patients to receive feedback and support from their family and friends. It should be noted here that, for instance, 63.3% of those surveyed live with a steady partner, and in the focus groups, it was frequently stated that sustained weight loss could only be achieved with the support or encouragement of a partner. In the preference analysis, social support lies in the middle of the preferences; it is, however, an essential component of successful sustained weight loss.

### Knowledge

Imparting of knowledge with regard to possibilities for weight loss is a necessary aid for patients to enable achievement of therapeutic success (coefficient: 0.8889). An important component of today's therapies is the transfer of knowledge and education. The guidelines of the German Obesity Society expressly recommend this therapy component (at least 10 hours of group teaching per rehabilitation period (in Germany between usually 19 and 21 days)) [45]. Teaching content is given as nutritional advice, cooking schools and exercise instruction. The transfer of information enables obese patients to organise their lives themselves and to acquire and implement knowledge about the necessary behavioural changes. Byrne (2002) points out that renewed weight gain is often initiated by stress situations. The active teaching of problem solutions and stress management strategies, such as relaxation techniques, can considerably reduce the risk of renewed weight gain [51]. Patients should be given knowledge to enable them to prevent renewed weight gain; the success already achieved should be stabilised over time [48].

### Varied range of therapy options available

The preferability of a therapy is determined only to a small extent by the varied range of therapy and leisure activities available (coefficient: 0.6368), i.e. the preferability of a programme for weight loss is only negligibly affected by the variety of the services offered therein. Thus, for instance, the individualisation of therapy is more important than the varied range of therapy measures available.

### Hotel and service aspect

In the preference evaluations of overweight and obese patients, the attribute of hotel and service aspects achieved the lowest coefficient and, therefore, was of least importance within the decision (0.5709). It can be assumed that patients are prepared to forego comfort and amenities in favour of coordinated, individualised and interpersonal therapy. Nevertheless, certain standards must be maintained in the rehabilitation facility (e.g. standards of cleanliness and service) and weight-adapted furnishings (e.g. appropriate chair sizes and reinforced beds and toilet fittings) are appreciated.

## Conclusion

On the one hand, the consideration and integration of the patient perspective during the design, implementation and evaluation is seen as an important success factor for integrated care programmes and, on the other hand, the guarantor for better treatment outcomes [52,53]. The first three attributes (coordination and referral, individualised therapy planning and interpersonal care) are describing 50% of the preferences (relative frequency) within the therapy-relevant attributes examined. It can be assumed that the offer of an optimally coordinated, individually adapted and interpersonal therapy process satisfies needs and expectations and increases patient compliance and adherence and, therefore, enables the achievement of sustained weight reduction.

This study focussed on the evaluation of therapy-based preferences within weight reduction therapy for overweight and obese rehabilitation patients. From the sequence of the attributes within the preference evaluation, it is clear that the behaviour-related characteristics are foremost. Academic literature expressly states that only long-term behavioural modification can enable achievement of sustained success. Therefore, continuous care, coordination of individualised therapy planning and interpersonal care are of exceptional importance. For this reason, current treatment guidelines (e.g. those of the German Obesity Society) and a number of therapy plans already contain preference-influencing dimensions and characteristics of patients as far as possible. This study emphasises the importance of these therapy characteristics from the patient's perspective.

In general, it can be inferred that intervention for weight reduction is a long, drawn-out treatment process. Within the scope of rehabilitation measures, it is really only possible to lay the foundations for a long-term success. This is because obesity is often not the primary cause of being in the rehabilitation facility, the condition is instead only being treated ‘on the side’ as a comorbidity. Thus, the length of stay is generally limited to the 19–21-day period prescribed by the statutory health insurers, and treatment of the obesity is not central to the therapy plan. Thus, it is not possible to achieve sustained weight loss. Since overweight and obesity are, however, risk factors of the primary indication in many conditions (e.g. coronary heart disease, metabolic disorders, conditions of the musculoskeletal system, and malignant growths) therapy of the condition is of great significance [1].

Thus it is even more important that a long-term and coordinated weight loss measure is initiated during rehabilitation in order to prevent further onset or chronification of comorbidities.

One important advantage in rehabilitation is that the patients are removed from their familiar domestic and social environment and are in the rehabilitation facility all day. This enables the preparation and initialisation of reflection on behaviour up to now (e.g. in group discussions), a definition of the problems and a strategy for weight loss. And rehabilitation also offers the chance for an individualised design of weight loss measures.

The direct and individual consideration of personal circumstances and the orientation of treatment towards patient preferences can thus ensure long-term motivation. What is important in this respect is to build on optimal behaviour and emphasise the consequences of not losing weight through knowledge transfer.

In addition, help should also be provided to make lifestyle changes possible and to support patients in the transition from rehabilitation facility to the domestic setting. This includes helping the patient participate in follow-up measures after rehabilitation. As can be seen from the results of this study, continuous and individualised care is essential from the point of view of the patient.

## Summary, limitations and options for further research

In the course of this study, we were able to identify the patient preferences of therapy aspects for sustained weight loss in rehabilitation. Coordination of therapy and referral, individualised therapy planning and also interpersonal care are of greatest significance. The study was also able to show that patients are prepared to forego hotel and service aspects if these are compensated by coordinated, individualised and competent intervention.

Despite the relatively small sample, the study could yield significant results. However, the sample was not representative and the results should be interpreted with this respect. It must be noted that the respondents were recruited purely from three regional rehabilitation facilities in the German Federal State of Mecklenburg-Vorpommern. Nevertheless, the catchment area of these facilities does cover the whole of Germany so that the patients surveyed were not solely from this region.

The discrete-choice method works with the model of a *representative evaluating person*, i.e. it is assumed that the persons participating in a survey are representative of the body of those whose preferences are to be ascertained (e.g. the overweight patient sample). An analysis of the choices yields the preferences of the ‘representative’ of the population surveyed. Nevertheless, there might be subgroup differences within the ‘representative’ sample (heterogeneity of individual needs and expectations). With a sufficiently large number of respondents, it would be possible (e.g. by way of cluster analysis) to identify subgroups which have largely homogenous, but in themselves heterogeneous, preferences. Within the scope of this research question, it would be possible to analyse, for instance, whether preferences differ between the individual body mass index clusters. But even in the case of (patient) group segmentation, the discrete choice method can only enable identification of representative preference structures of a group or subgroup. In this case with overweight and obese patients, for instance, segmentation according to family status and the various preferences in this regard would be a possibility. In this study, the extent to which the therapy preferences differ within the individual subgroups remains open. It may be useful to distinguish by gender, family status, body mass index classification or other socio-demographic characteristics. In addition, the extent to which expectation of results and the possible reduction of risk affect preferences in regard to sustained weight loss programmes could be of interest, but the low sample size did not allow a robust estimation.

These results serve as a preliminary trial for a preference-based documentation of the motivations of rehabilitation patients. With a larger population, it would be possible to analyse heterogeneous preference structures and to identify the various requirements of an intervention in the future.

Knowledge of patient preferences enables influence to be exerted upon motivation, which is the starting point for any change of behaviour. Further investigation is required, however, to identify the precise interactions and causalities. The results of this study can, however, be used to illustrate the preference-weighted satisfaction of patients in rehabilitation. This knowledge can be used in quality management and in the continuous process of improving the quality of rehabilitation therapy measures in terms of their structure, process and results.

Therapy concepts and treatment processes must maximise patient utility in the long term. This orientation is lacking since there are hardly any studies on patient preferences or the expectations of sustained lifestyle changes in rehabilitation. The objective for the future must be to gain an insight into the individual preference structures of overweight and obese patients; only then will it presumably be possible to motivate patients to long-term behavioural change.

## Figures and Tables

**Table 1.**
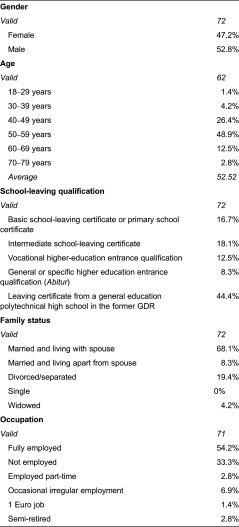
Socio-demographic characteristics of discrete-choice experiment participants

**Table 2.**
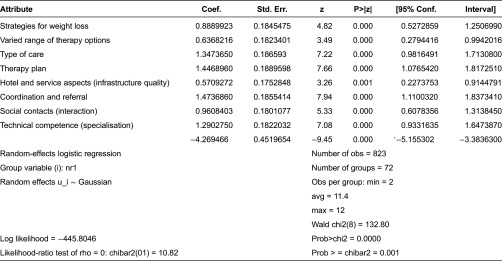
Coefficients of logit calculation
